# Whokaryote: distinguishing eukaryotic and prokaryotic contigs in metagenomes based on gene structure

**DOI:** 10.1099/mgen.0.000823

**Published:** 2022-05-03

**Authors:** Lotte J.U. Pronk, Marnix H. Medema

**Affiliations:** ^1^​ Bioinformatics Group, Wageningen University, Wageningen, The Netherlands

**Keywords:** metagenomics, taxonomy, gene structure, machine learning, biosynthetic gene cluster

## Abstract

Metagenomics has become a prominent technology to study the functional potential of all organisms in a microbial community. Most studies focus on the bacterial content of these communities, while ignoring eukaryotic microbes. Indeed, many metagenomics analysis pipelines silently assume that all contigs in a metagenome are prokaryotic, likely resulting in less accurate annotation of eukaryotes in metagenomes. Early detection of eukaryotic contigs allows for eukaryote-specific gene prediction and functional annotation. Here, we developed a classifier that distinguishes eukaryotic from prokaryotic contigs based on foundational differences between these taxa in terms of gene structure. We first developed Whokaryote, a random forest classifier that uses intergenic distance, gene density and gene length as the most important features. We show that, with an estimated recall, precision and accuracy of 94, 96 and 95 %, respectively, this classifier with features grounded in biology can perform almost as well as the classifiers EukRep and Tiara, which use k-mer frequencies as features. By retraining our classifier with Tiara predictions as an additional feature, the weaknesses of both types of classifiers are compensated; the result is Whokaryote+Tiara, an enhanced classifier that outperforms all individual classifiers, with an F1 score of 0.99 for both eukaryotes and prokaryotes, while still being fast. In a reanalysis of metagenome data from a disease-suppressive plant endospheric microbial community, we show how using Whokaryote+Tiara to select contigs for eukaryotic gene prediction facilitates the discovery of several biosynthetic gene clusters that were missed in the original study. Whokaryote (+Tiara) is wrapped in an easily installable package and is freely available from https://github.com/LottePronk/whokaryote.

## Data Summary

The authors confirm that all supporting data, code and protocols have been provided within the article or through supplementary data files.

Whokaryote was developed in Python 3 as a command-line application for Linux, macOS and Windows.

The source code and documentation are available on GitHub under a GPLv3 licence (https://github.com/LottePronk/whokaryote).

The datasets that were used to train and test Whokaryote were constructed from National Center for Biotechnology Information (NCBI) reference genomes. GenBank accession numbers are listed in Supplementary Material S1–S3 (available in the online version of this article). The FASTA and GFF files used are available from https://git.wageningenur.nl/lotte.pronk/whokaryote/-/tree/master/train_test_files.

**Impact Statement**

Studying the collective genomes of complete microbial communities (known as metagenomes) can provide important insights into microbial ecology. Most studies and, consequently, analysis pipelines, focus on the bacterial members of these communities, while ignoring eukaryotes. Because eukaryotic sequences require eukaryote-specific analysis pipelines, it is necessary to separate prokaryotic from eukaryotic contigs at an early stage of data processing. Homology-based tools can perform taxonomic classification down to the species level, but they use databases in which eukaryotes are often underrepresented. Other tools use k-mer frequencies to classify sequences on the eukaryote/prokaryote level without the need of databases, but it is unknown what biological features these k-mers represent. With Whokaryote, we use features based on fundamental differences between prokaryotes and eukaryotes in terms of gene structure, and optionally combine these with k-mer-frequency-based Tiara predictions to achieve highly accurate classification of metagenomic contigs. Standalone, Whokaryote is fast and performs almost as well as other tools. The combined classifier Whokaryote+Tiara outperforms all tested individual classifiers in terms of accuracy. Repredicting genes on eukaryotic contigs identified as such by Whokaryote+Tiara results in better downstream functional annotation, aiding the discovery of ecological functions of eukaryotes in microbiomes.

## Introduction

Microbiomes are increasingly recognized for playing a large role in the health and development of their hosts [1, 2]. Studies aiming to characterize these microbial communities are increasingly shifting from largely marker gene-based community abundance profiling (e.g. 16S, ITS) to analyses of the complete metagenome using shotgun sequencing. Analysing the complete metagenome has provided valuable insights into microbial ecology and microbiome–host interactions [3–5].

Many metagenomic analysis pipelines assume that all contigs will be prokaryotic, while eukaryotic micro-organisms are also present in microbiomes [6–9]. Including eukaryotes in metagenome analyses could provide a more complete picture of their ecological role and functional capabilities. To study eukaryotic sequences in metagenomes, a reliable taxonomic classification method at the contig level is required.

Most current metagenome taxonomic classification tools were largely designed with prokaryotes in mind. Tools such as CAT/BAT [10], DIAMOND+MEGAN [11], or Kraken2 [12] are good at assigning taxonomy to metagenomic contigs up to the species level, but they require relatively time-consuming sequence homology searches (CAT, DIAMOND+MEGAN) or use k-mers (Kraken2) and thus require the use of large pre-existing databases. Uncultivated organisms are frequently missed because they are not yet in a database. Additionally, some sequence homology-based methods, such as CAT, require accurate gene predictions, which paradoxically require knowledge of at least the empire (eukaryote/prokaryote) level for each contig to select the best gene predictor. Prokaryotic gene predictors do not consider introns, making them unsuitable for predicting eukaryotic genes. The eukaryotic gene predictor Augustus [13] uses models that were trained on specific organisms and can be trained on metagenomic bins to predict the genes in such a bin, an approach used in the EukRep pipeline [14]. However, there is no good solution for *de novo* gene prediction in unbinned contigs. MetaEuk predicts protein-coding genes on eukaryotic metagenomic contigs, but relies on a large and likely incomplete reference database of protein profiles [15]. Because of the limitations of current methods, many metagenomic studies may be missing eukaryotic genes altogether because they annotate them incorrectly. K-mer-based methods such as Kraken2 do not rely on gene predictions, but they require a database of closely related reference genomes. Not many reference genomes of eukaryotic micro-organisms are available in public databases yet, which could lead to an underestimation of eukaryotes in microbiomes.

In order to allow metagenomic analysis pipelines to selectively run eukaryote-specific gene finding on all contigs likely to be eukaryotic (whether belonging to a known taxon or not), simply classifying metagenomic contigs as either prokaryotic or eukaryotic (instead of comprehensively assigning taxonomy) would be a logical solution. Some efforts have recently been made in this direction. For example, EukDetect maps reads to a database of universal eukaryotic marker genes to detect eukaryotes in metagenomes [16], but this approach will not find contigs that do not contain these marker genes. The tool EukRep [14] calculates k-mer counts of 5 kb fragments of (meta)genomic contigs and then classifies the complete contig based on a majority vote using a support vector machine model. The authors show that using eukaryotic gene predictors on sequences classified as eukaryotic leads to more accurate annotations and downstream analyses, including higher-level taxonomic classification [14]. Despite its high overall accuracy, EukRep does not perform very well on several prokaryotic and eukaryotic taxa, especially parasites and symbionts. Moreover, it was trained and tested on relatively long contigs, while metagenome assemblies often consist of mostly short contigs. More recently, Tiara, a classifier that uses k-mer counts as features for deep learning models was published [17], which was shown to be slightly more accurate than EukRep. Tiara was developed with detecting organelle sequences in mind, and therefore has a more fine-grained classification, distinguishing archaea, bacteria, organelles and eukaryotes. Like EukRep, the performance of Tiara is less accurate on certain organisms. Because the approaches used by both tools do not allow for the inference of feature importance, it is very difficult, if not impossible, to determine which k-mers are important for distinguishing between eukaryotes and prokaryotes, and what underlying biological features these k-mers represent. This makes it difficult to correct for any biases. Moreover, k-mer-based approaches by their nature likely require training data from closely related taxa to work well, making it probable that contigs from uncultivated taxa unique to specific biomes will be misclassified.

Here, we introduce Whokaryote, a random forest classifier with comparable performance to previous k-mer-based approaches, but which uses manually selected features based on fundamental differences in gene structure between eukaryotes and prokaryotes. Whokaryote uses intergenic distance, gene density and other genomic features to predict whether a given metagenomic contig belongs to a eukaryote or a prokaryote. Despite the different approach, our tool performs as well and, in some cases, better than EukRep and Tiara on contigs with sizes often found in metagenomes. We also show that using eukaryote-specific tools to analyse the classified eukaryotic contigs can lead to the discovery of microbial traits that have remained undiscovered when solely using prokaryote-oriented tools. Finally, by using Tiara predictions as an additional feature, we constructed a highly accurate classifier with an F-1 score of 99.2 % on a simulated metagenomic dataset. With this enhanced classifier, which we term Whokaryote+Tiara, sequences can be classified reliably for a very wide range of eukaryotes and prokaryotes.

## Methods and implementation

### Training dataset for the random forest classifier

To train the random forest classifiers, we downloaded 73 prokaryotic (68 bacteria and 5 archaea) genome assemblies from 31 different phyla, and 25 eukaryotic (12 fungi, 10 protists, 1 plant and 2 animals) genome assemblies from 14 different phyla from the National Center for Biotechnology Information (NCBI) GenBank database (see Supplementary Material S1). Despite the relatively small number of genomes included, we think the diversity of phyla and the fact that we split genomes into numerous shorter fragments provides enough data to train a sufficiently generalized model. All non-genomic DNA sequences were removed using the following search terms: API, MIT, mitochondrial, plastid, chloroplast, mitochondrion, non-nuclear, organelle and apicoplast. Next, these genomes were split into non-overlapping artificial contigs with a random length of 5000–100 000 base pairs according to a triangular distribution with the lower left limit at 5000, the mode at 10 000 and the upper right limit at 100 000 bp. For the training set, a maximum of 500 contigs per genome were used to prevent an overrepresentation of eukaryotic contigs, which tend to have very large genomes compared to prokaryotes. For every contig (both prokaryotic and eukaryotic), genes were predicted using the prokaryotic gene prediction tool Prodigal [18] (version 2.6.3) using the metagenomic setting (--meta). We used Prodigal because it is already commonly used in metagenomics pipelines and preparing these output files as input to our classifier will save time in the subsequent gene prediction step. Contigs with one predicted gene or no predicted genes were excluded in further steps (see below for explanation). This resulted in a dataset with a total of 11 285 eukaryotic contigs and 8403 prokaryotic contigs that we used to train and validate the random forest classifier.

### Used features

Genes in prokaryotes are often packed closely together on the genome in co-regulated operons that are transcribed to a single polycistronic mRNA. Therefore, we expected genes on eukaryotic contigs to generally have a higher intergenic distance and a lower gene density than prokaryotic contigs. Additionally, because genes in operons are under the control of a single promoter, we expected that adjacent pairs of bacterial genes on a given stretch of the genome would have a higher probability of being present on the same strand, i.e. they would have the same orientation. Because calculation of our selected features requires the presence of at least two genes, contigs with only one predicted gene or no predicted genes are excluded from our classification.

For each contig, the intergenic distance of each gene pair was calculated (start position gene 2 − end position gene 1=intergenic distance). Next, the mean, standard deviation, and the first and third quartile of the intergenic distance per contig were calculated and used as features for the classifier. The length of every gene was calculated by subtracting the stop position from the start position. The gene density was calculated by dividing the sum of the length of every gene on a contig by the total length of the contig.

In the prodigal gene location output file, genes that are on the positive strand are annotated with a ‘+’ sign, and the genes on the negative strand are annotated with the ‘−’ sign. For every contig, the ratio of genes that are on the same strand was calculated as follows. We determined for every adjacent pair of genes if they are located on the same strand (++ or −−) or not. The number of pairs that were located on the same strand was divided by the total number of gene pairs. The outcome was used as a feature for the classifier. Finally, the mean gene length of every contig was calculated and used as a feature.

Prodigal also predicts whether a ribosome-binding site (RBS) motif is present upstream of a gene. Because these motifs are prokaryote-specific, we expected that on average, Prodigal would find such RBS motifs more frequently on prokaryotic contigs than on eukaryotic contigs. Therefore, we calculated the ratio of RBS motifs per gene present on each contig, and used this as a feature in our model.

In our enhanced classifier, we used Tiara predictions as an additional feature for training and testing. Therefore, we named this classifier Whokaryote+Tiara. To obtain Tiara predictions, we used Tiara (version 1.0.2), using the DNA sequence of the contigs as input, and setting the minimum contig length to 3000. We converted them into usable features by converting the predictions to a number: 0 for contigs classified as ‘eukarya’; 1 for contigs classified as ‘prokarya, ‘bacteria’ and ‘archaea’; 2 for contigs classified as ‘unknown’ or ‘organelle’.

Features were also calculated for the reference genome annotations downloaded from the NCBI and mapped to the corresponding artificial contigs so the values could be compared to the prodigal annotations.

### Random forest classifier

Two different classifiers were trained, one without Tiara predictions as a feature, which we named Whokaryote, and one with Tiara predictions as a feature, which we named Whokaryote+Tiara. All features were stored in a dataframe with contigs as rows and features as columns. To build the random forest classifier, we used the Python package scikit-learn version 0.23.2 [19].

A random forest with 100 estimators was initialized with parameter *class_weight* = ‘balanced’, which applies weights to each class to correct for the class imbalance of the dataset. Next, fivefold cross-validation was used with the function *StratifiedGroupKFold*, with the organism names as the ‘group’ parameter to prevent contigs from the same genome/organism being present in both the training and test data for each fold. Additionally, for each fold, class labels are stratified to maintain the proportion of class labels from the original dataset. Each contig was labelled as ‘eukaryotic’ or ‘prokaryotic’. The function *cross_validate* was used to calculate the accuracy, precision and recall for each of the five folds. The final model was trained on the complete dataset and was tested on a separate dataset containing contigs from different organisms, including those from phyla that were not present in the training dataset. Feature importance was calculated using the *feature_importances* function.

### Code availability

The standard random forest classifier and the enhanced classifier have been made available in a downloadable Python package that can be used on the Linux command line. The package and instructions to install it are available at https://github.com/LottePronk/whokaryote.

## Results and discussion

**Fig. 1.**
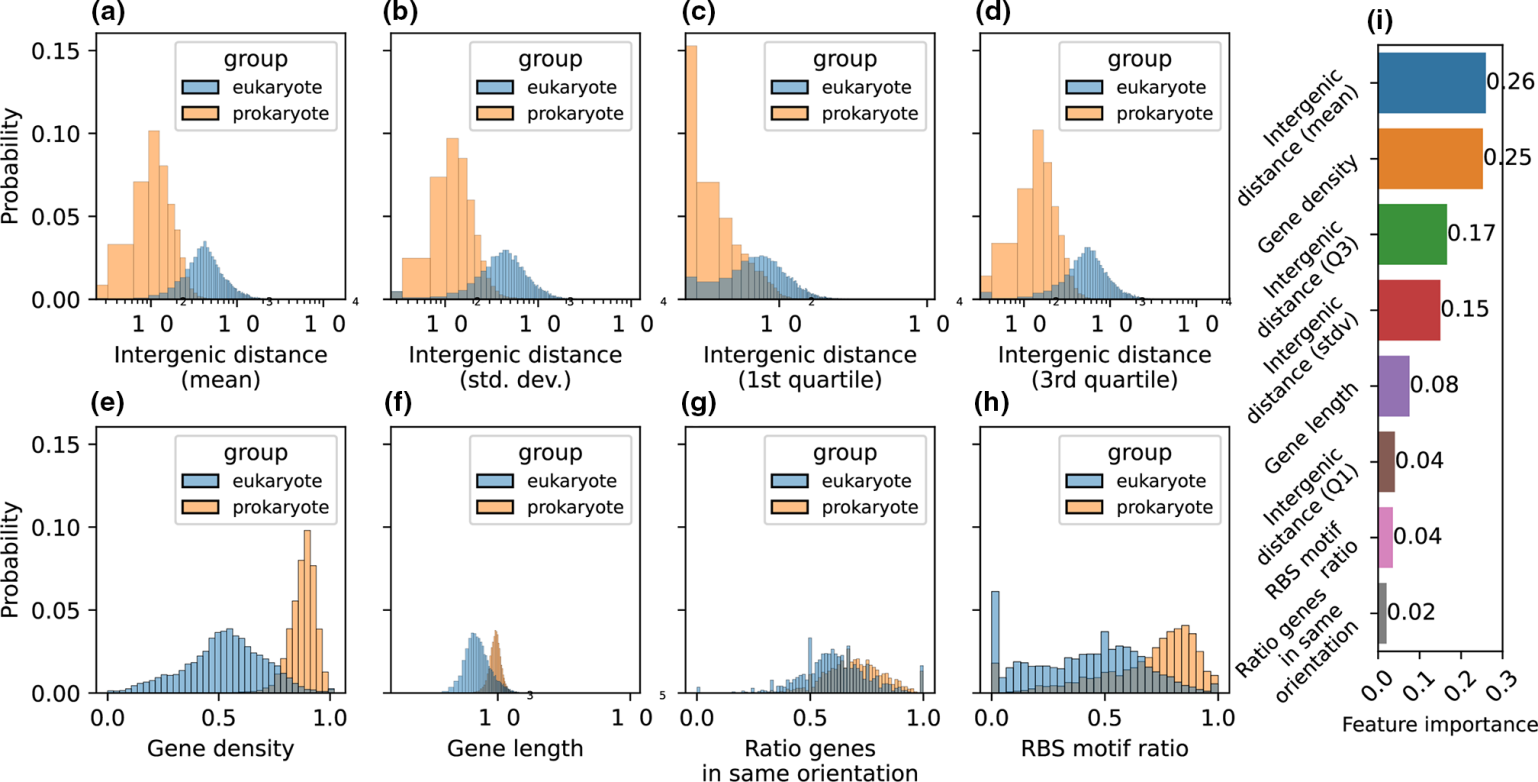
Statistical overview of the features calculated for the training and validation dataset containing gene-based features of 18 708 artificial contigs taken from 73 prokaryotic and 25 eukaryotic reference genomes. Gene prediction was performed using Prodigal-M. The probability distribution of the feature values of all contigs are shown, grouped by the taxonomic group (eukaryote or prokaryote) the contig belongs to. For every contig, the mean, standard deviation, first quartile and third quartile of the intergenic distances between the genes was calculated [(a(–(d), respectively]. (e) The mean gene density of every contig, calculated by dividing the sum of the length (in base pairs) of all genes by the total length of the contig. (f) The mean gene length of every contig, calculated as the end position of the gene minus the start position. (g) The ratio of genes on a contig that are on the same strand (e.g. they have the same ‘orientation’). (h) The ratio of ribosome-binding site (RBS) motifs predicted by Prodigal to the total number of genes per contig. (h) The importance of each feature that was used in our random forest classifier trained on artificial contigs of 99 different organisms. (i) The importance of each feature that was used in our random forest classifier trained on artificial contigs of 99 different organisms.

First, we made a random forest classifier that determines whether a metagenomic contig (or any genomic sequence) is of eukaryotic or prokaryotic origin by using features based on fundamental differences between the genome structures of prokaryotes and eukaryotes. To train our classifier, we used a dataset of 73 prokaryotic and 25 eukaryotic reference genomes that were fragmented into shorter artificial contigs to resemble a metagenome assembly. Prodigal (metagenomic mode) was used to predict genes on these contigs, and the resulting gene coordinates file was used to calculate our chosen features. Per contig, we calculated the intergenic distance (average, first quartile, third quartile and standard deviation), gene length (average), gene density, the percentage of gene pairs with the same orientation, and the ratio of RBS motifs to total number of genes. As expected, the intergenic distance was lower on bacterial contigs than on eukaryotic contigs (Fig. 1a–d). The gene density was higher on prokaryotic contigs compared to eukaryotic contigs (Fig. 1e) and is likely linked to the intergenic distance. The gene length was higher for prokaryotes (Fig. 1f). This might be viewed as unexpected, since eukaryotes have introns that can make up a large part of the total gene length; however, we used Prodigal to predict genes on the contigs, which does not consider introns and may predict a gene for every exon, often further reduced in length because the first start codon of an exon may be considerably downstream of the intron/exon junction. Lastly, the percentage of gene pairs on a contig that are in the same orientation was only slightly higher in prokaryotes (Fig. 1g), but we still used it for training our classifier. Finally, because Prodigal predicts specific prokaryotic RBS motifs, we expected it to find fewer of those motifs on eukaryotic contigs. Our results show that Prodigal does find RBS motifs on eukaryotic contigs, but (on average) fewer than on prokaryotic contigs (Fig. 1h). It is likely that these motifs are not true RBSs, but just sequences that are incidentally identical to prokaryotic RBS motifs, which may happen because these motifs are fairly short.

The features we calculated are based on non-ideal gene predictions for eukaryotes, and we wanted to know if the values for each feature we observed are representative and can be used to make claims about biological differences. Therefore, we also calculated the same features for the reference annotations that we mapped to the artificial contigs. Indeed, all features, except for gene length, show (almost) the same distributions in the reference annotations for both eukaryotes and prokaryotes as in the features we calculated with the Prodigal annotations (Fig. S1). According to the reference annotations, eukaryotes have longer genes than prokaryotes, as is expected because of their introns.

After calculating the features, we initialized a random forest with 100 estimators and used fivefold cross-validation to estimate its performance. On average, the model had an accuracy of 95.2 %, a precision of 94.1 % and a recall of 96.0 %.

We trained our final model on the entire dataset and calculated the feature importance. The average intergenic distance, gene density and the third quartile of the intergenic distance were the most important features, whereas the ratio of genes in the same direction and the ratio of RBS motifs are less important (Fig. 1h). Prodigal annotations are thus suited for our classification goal, but it is important to keep in mind that the classifier is based on differences in features that arise partly from inadequately annotating eukaryotic contigs. Nevertheless, the differences in features we see between eukaryotes and prokaryotes (except for gene length) are in line with current knowledge of the gene structures of these organisms, and the importance of those features to our classifier supports this.

### Whokaryote vs EukRep and Tiara

Because we wanted to make sure that our classifier, which we call Whokaryote, is also accurate on organisms that were not in our training set, we ran our classifier on another test dataset from the genomes of 14 eukaryotes from 8 different phyla and 25 prokaryotes from 19 different phyla (only 2 phyla of which were also present in the training dataset) (Supplementary Material S2), for which we prepared 7034 artificial contigs in the same way as with the training dataset. This resulted in 4848 eukaryotic contigs and 2186 prokaryotic contigs. Most of the eukaryotic organisms in this dataset were also not used in the training data for EukRep and Tiara (Supplementary Material S2). The accuracy of Whokaryote on this dataset was 95 %, with a precision and recall of 0.97 and 0.96 for eukaryotes, and 0.92 and 0.93 for prokaryotes, respectively (Table 1). We also wanted to know how our classifier performs in comparison to EukRep and Tiara, which use k-mer counts of fragmented contigs as features for a support vector machine classifier and a neural network-based classifier, respectively. We ran our classifier, EukRep (default model, chunk size 5000 bp) and Tiara (default settings) on our test dataset to compare their performance. EukRep had an overall accuracy of 96 %, and for prokaryotic contigs a precision of 0.91 and a recall of 0.99. For eukaryotic contigs, it had a precision and recall of 0.99 and 0.95, respectively (Table 1). Tiara had an overall accuracy score of 0.98, a precision and recall of 1.00 and 0.97 for eukaryotes, and a precision and recall of 0.98 and 1.00 for prokaryotes (Table 1). It also identified sequences as ‘unknown’ or ‘organelle’, while we excluded organellar DNA from our dataset.

**Table 1.** Accuracy metrics for contig_classifier, EukRep, Tiara and Whokaryote on a test dataset of 7034 artificial contigs (sizes between 5100 kbp, mean length 34684 bp) taken from the reference genomes of 14 eukaryotes and 25 prokaryotes

	Whokaryote			EukRep			Tiara			Whokaryote+Tiara			
	**P**	**R**	**F1**	**P**	**R**	**F1**	**P**	**R**	**F1**	P	R	F1	Support
**Eukaryote**	0.97	0.96	0.97	0.99	0.95	0.97	1.00	0.97	0.98	0.99	1.00	0.99	4848
**Prokaryote**	0.92	0.93	0.93	0.91	0.99	0.95	0.98	1.00	0.99	0.99	0.99	0.99	2186
**Organelle**	–	–	–	–	–	–	0.00	0.00	0.00	–	–	–	0
**Unknown**	–	–	–	–	–	–	0.00	0.00	0.00	–	–	–	0
													
**Accuracy**			0.95			0.96			0.98			0.99	7034
**Macro average**	0.94	0.95	0.95	0.95	0.96	0.96	0.50	0.49	0.50	0.99	0.99	0.99	7034
**Weighted average**	0.95	0.95	0.95	0.97	0.96	0.96	0.99	0.98	0.98	0.99	0.99	0.99	7034

F1, F1 score; P, precision; R, recall.

We then wanted to know whether all three classifiers make similar mistakes. Firstly, we calculated the accuracy per bin of contig lengths with bin sizes of 10 kbp. Whokaryote and EukRep are less accurate on contigs with a length between 5 and 20 kbp, but with at least 90 % of contigs classified correctly, the accuracy is still relatively high (Fig. S2). Tiara shows a more consistent performance across all contig lengths, although predictions on contigs shorter than 20 kbp also tend to be slightly less accurate. For our classifier, the lower accuracy may stem from the fact that shorter contigs contain fewer genes based on which the features are calculated. The EukRep classifier may be less accurate on shorter contigs because these can be fragmented into fewer 5 kbp pieces than longer contigs, making a majority vote to determine the overall contig class less reliable. It is thus recommended to use classification tools on contigs of at least 5 kbp.

**Fig. 2.**
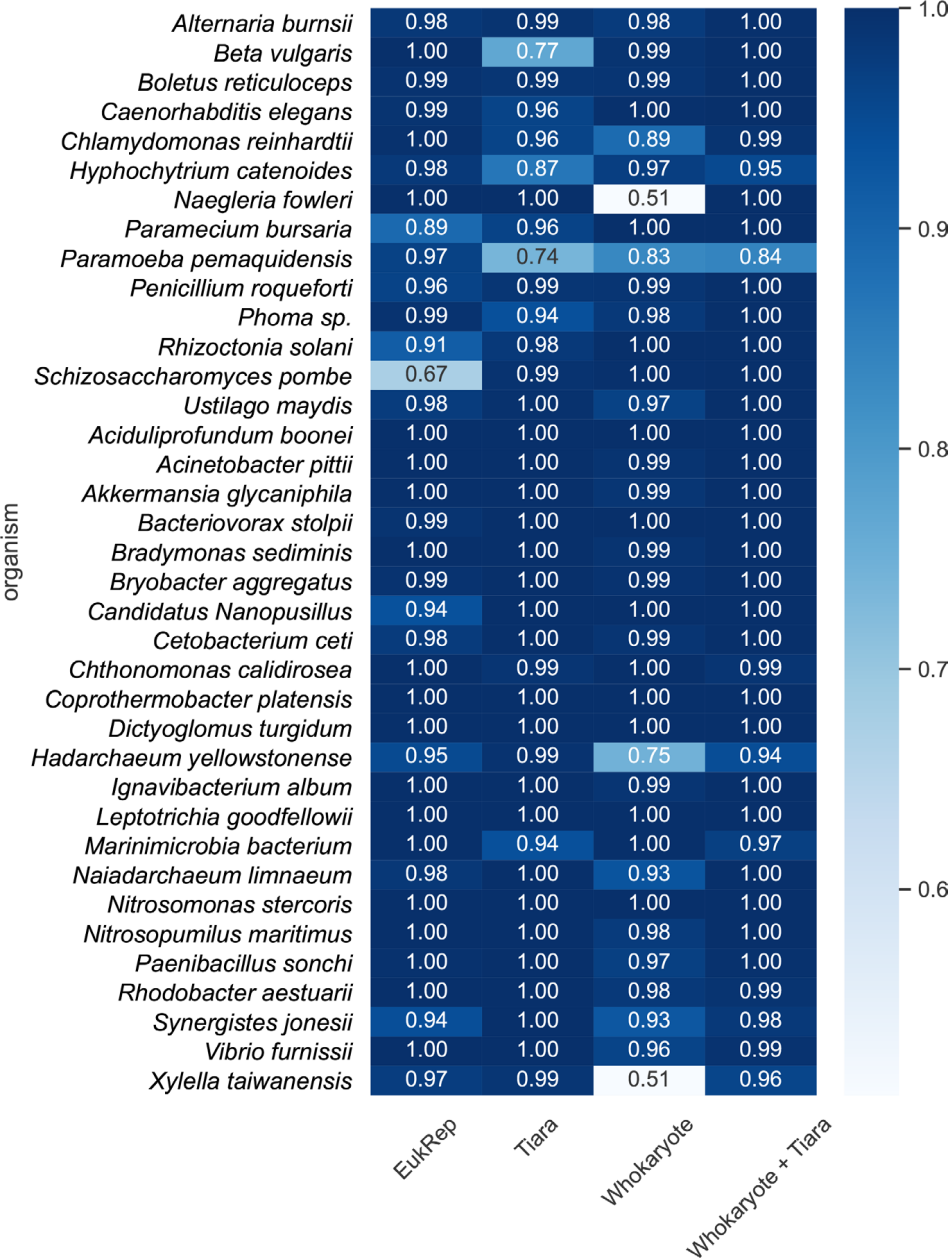
Comparison of the accuracy (depicted as relative count of incorrect and correct predictions) per organism of EukRep, Tiara, Whokaryote and Whokaryote+Tiara on a test dataset consisting of 7034 artificial contigs from 14 eukaryotes and 25 prokaryotes.

When we calculate the accuracy on contigs per organism, we see that each classifier has problems with different organisms (Fig. 2). Whokaryote makes the highest number of mistakes on contigs from the eukaryote *Naegleria fowleri*, which is an amoeba with a relatively high gene density (Fig. S3), within a range that is more typical for prokaryotes. Because gene density is an important feature of Whokaryote, contigs from organisms with many genomic regions that fall outside the normal range of gene density for their class are more likely to be wrongly classified. Compared to EukRep and Tiara, Whokaryote is also less accurate on *Chlamydomonas reinhardtii* contigs, which also have a relatively high gene density, with a few outliers that lie within the gene density range of most prokaryotes. Interestingly, EukRep performed well on *Paramoeba pemaquidensis* contigs, and Tiara, Whokaryote and Whokaryote+Tiara performed worse. This is the other way around for contigs of *Paramecium bursaria*, *Rhizoctonia solani* and *Schizosaccharomyces pombe*. This may indicate that the k-mers that EukRep picked up as important features are different than those that Tiara picked up and are also not related to Whokaryote features. (Fig. 2). It is remarkable that EukRep performed relatively poorly on *S. pombe*, because it was trained on this organism. The EukRep training genomes were not fragmented as much as those in our training set, however. If distinct k-mer frequencies are unevenly distributed across the genome, the distributions in larger contigs may be different than in shorter contigs. This may be a limitation of using k-mer counts as the only features. Tiara performed relatively poorly on *Beta vulgaris*, a higher plant. This may be because Tiara was not trained on higher plant nuclear genomes, but only on green algae. On the other hand, EukRep and Tiara performed better on *Xylella taiwanensis* contigs, with ~95 % of contigs classified correctly, versus only ~50 % by Whokaryote (Fig. 2). We expected that our model would be less accurate in predicting the class of a contig when the genome structure of the organism was atypical and more resemblant of the other class. Indeed, approximately half of the contigs of *X. taiwanensis* have an intergenic distance and gene density that fall within a range that is closer to that of most eukaryotes (Fig. S3). This is in line with reports of *X. taiwanensis* having many pseudogenes [20], which are relatively common in pathogenic bacteria; the likely cause of this is a reduced effective population size during host infection, which causes mutations to accumulate [21]. We use a limited set of features that are all related, which means that if the gene structure in (part of) an organism’s genome is atypical, the classifier may not recognize this and assign the wrong class.

All three classifiers are based on different machine learning methods and features, which have their own advantages and drawbacks. For both EukRep and Tiara, it is unknown which k-mers are the most important for classification and if they correspond to any known biological signal. Our classifier makes use of manually selected features based on prior biological knowledge and on its own performs (almost) equally well as the other classifiers. However, all three tools make different mistakes, indicating that the k-mers represent sequence motifs that do not necessarily correspond to our features. We speculate that gene structure-based features may be more general because of the fundamental biological differences between eukaryotes and prokaryotes. Methods that use k-mer frequencies may pick up on signals, such as specific sequence motifs, that are more specific to higher taxonomic ranks and may be more accurate on genomes that are closely related to training set sequences. A very wide training database, such as those used by Tiara and EukRep, may alleviate this problem. Our relatively small training dataset and the positive results on our test dataset confirm that our features are also predictive for genomes more distantly related to those of the training set.

### An enhanced classifier that uses Tiara predictions as an additional feature is highly accurate

Because all three classifiers make mistakes in different contigs, it is possible that the important k-mers of EukRep and Tiara contain information that is complementary to the features based on gene structure that we have used. Therefore, we hypothesized that if we included the Tiara output as a feature to retrain our own classifier, the overall accuracy should improve. Indeed, retraining the classifier with the Tiara output from the first step, with ‘bacteria’ and ‘archaea’, and ‘unknown’ and ‘organelle’ collated into single classes, resulted in a classifier with an overall accuracy of 99.2 %. The rounded F-1 scores are 0.99 for both eukaryotes and prokaryotes (Table 1).

The Tiara prediction was the most important single feature, with a score of 0.38, while gene density and intergenic distance in sum had a somewhat larger feature importance (with 0.21 and 0.20, respectively) (Fig. S4). The enhanced classifier improves the accuracy scores for the organisms that either of the individual classifiers performed weakly on, such as *Xylella fastidiosa* (Fig. 2). This shows that by combining two classifiers with different feature types, the weaknesses of each classifier can be compensated, which results in a highly accurate new classifier. We call the resulting classifier Whokaryote+Tiara.

The Tiara preprint reported a relatively high percentage of misclassifications in genomes from specific microbes with reduced genomes such as symbionts and parasites. We selected a set of such genomes to see if our initial classifier and Whokaryote would perform better. The genomes were divided into contigs of >5000 bp, resulting in +/− 1600 contigs in total, varying between 8 and 495 contigs per organism (Supplementary Material S3). On these contigs, we ran Tiara (standalone), Whokaryote and Whokaryote+Tiara (Fig. S5). The overall accuracy scores were 74 % for Tiara, 82 % for Whokaryote and 87 % for Whokaryote+Tiara. Whokaryote performed better than Tiara on 7 out of 10 organisms, and similarly on the other three organisms. Interestingly, Whokaryote performed better than both Tiara and Whokaryote+Tiara on four organisms. Tiara performed the worst on the eukaryotic microbe *Cafeteria roenbergensis* and on the bacterium *Mycoplasma haemofelis *, with <25 % of contigs classified correctly. Whokaryote+Tiara performed better on the contigs of these genomes, with +/− 76 and 33 % of contigs classified correctly, respectively. With 56 % correctly identified contigs, Whokaryote performed better on *M. haemofelis* than Whokaryote+Tiara, which only classified 33 % of the contigs correctly. For the *Parcubacteria* metagenome-assembled genome, Whokaryote classified 100 % of the artificial contigs correctly, while Tiara and Whokaryote+Tiara classified 79 and 84% correctly, respectively. This indicates that in some cases the addition of the Tiara prediction as a feature leads to worse performance of Whokaryote+Tiara. When working with microbiomes that contain genomes of unusual (e.g. parasitic) organisms, it may therefore be advisable to (also) use the standalone Whokaryote classifier.

### Comparison with a homology-based approach on a real-world dataset

To test whether our enhanced classifier also works on real-world data, we ran it on a real metagenome dataset of the endophytic root microbiome of sugar beet [3]. We ran metaProdigal on the metagenomic contigs >5 kbp to use as input for our classifier, together with the DNA sequences of the contigs for the Tiara classification. A total of 29 512 contigs with more than 1 gene, totaling 487 Mbp, were classified using a single core. The Tiara classification step took 281.6 s, and the rest of the classification took 114.8 s, resulting in a total time of 321.9 s (~5 min). The classifier predicted 1644 eukaryotic contigs and 27 868 prokaryotic contigs. We wanted to know whether these classifications were reliable and compared them to the classification of homology-based classification tool CAT [10] (standard settings, -n 20, nr database version 2021-01-07), which can taxonomically classify contigs up to the species level, but which took a much longer time, approximately 6 h and 49 min, to run (excluding gene prediction) on 20 cores. However, we only looked at the classifications on the superkingdom level, and we labelled the classifications ‘bacteria’ and ‘archaea’ as ‘prokaryote’, and ‘eukaryota’ as ‘eukaryote’. Out of 29 512 contigs, 28 806 (97.55 %) were classified with the same taxonomic label by both our classifier and CAT. Of the 706 contigs that did not have a matching classification, 163 were classified as ‘not_assigned’ by CAT, 351 were classified as ‘no support’ and 6 contigs were classified as viruses. When CAT cannot find a match for an ORF in a database, it cannot assign any taxonomic information to the contig, and therefore classifies it as ‘no support’ or ‘not_assigned’. With our classifier, we were able to classify these contigs as having a prokaryotic or eukaryotic origin. Of the remaining non-matching classifications, CAT classified 128 contigs as prokaryote, while our classifier identified them as eukaryote, and 58 contigs were classified as eukaryote by CAT and as prokaryote by our classifier. All in all, these results show that our enhanced classifier is also very accurate on real metagenomic data, and can quickly determine the portion of eukaryotic sequences, which can then be processed with eukaryote-specific tools.

### More fungal biosynthetic gene clusters detected on fungal contigs with repredicted genes

EukRep already showed that binning of eukaryotic contigs helps to improve gene predictions and functional annotations. However, there will always be contigs that cannot be binned. These may still contain interesting genes that may help explain the functions of the microbiome. The secondary metabolites/natural products that are produced by microbes are of special interest in this regard, as these molecules are often used to interact with other microbes and the environment. The biosynthetic gene clusters (BGCs) encoding the production of these molecules can be predicted by tools such as antiSMASH [22], which uses specific parameters and models for predicting bacterial BGCs, and a different set of parameters and models to predict fungal BGCs. Additionally, the bacterial version uses Prodigal as a gene finder, and the fungal version (fungiSMASH) uses GlimmerHMM [23], an *ab initio* eukaryotic gene finder. Currently, many metagenomic studies do not distinguish between fungal and bacterial contigs and run the bacterial version of antiSMASH on all contigs. Important fungal BGCs may be missed because of wrong gene annotations and the use of bacteria-specific models. We wanted to know if prefiltering metagenomic contigs into eukaryotic and prokaryotic could be a useful approach to select contigs that should (also) be run using the fungal mode of antiSMASH. We ran both the bacterial version (options --genefinding-tool prodigal-m) and the fungal version (options --taxon fungi, --cassis, --genefinding tool glimmerhmm) of antiSMASH on the contigs of our test dataset and compared the results (Supplementary Material S4). The bacterial version found 141 regions, of which 66 were located on eukaryotic contigs and 75 on prokaryotic contigs. The fungal version found 92 regions, of which 71 were located on eukaryotic contigs and 21 on prokaryotic contigs. Twenty eukaryotic BGCs were found by fungiSMASH that were not found by antiSMASH, showing that using eukaryote-specific tools on contigs classified as eukaryotic can lead to the discovery of functions that are missed when using prokaryote-specific pipelines to study metagenomes. Interestingly, bacterial antiSMASH also found BGCs on 15 eukaryotic contigs that were not found by fungiSMASH.

**Fig. 3.**
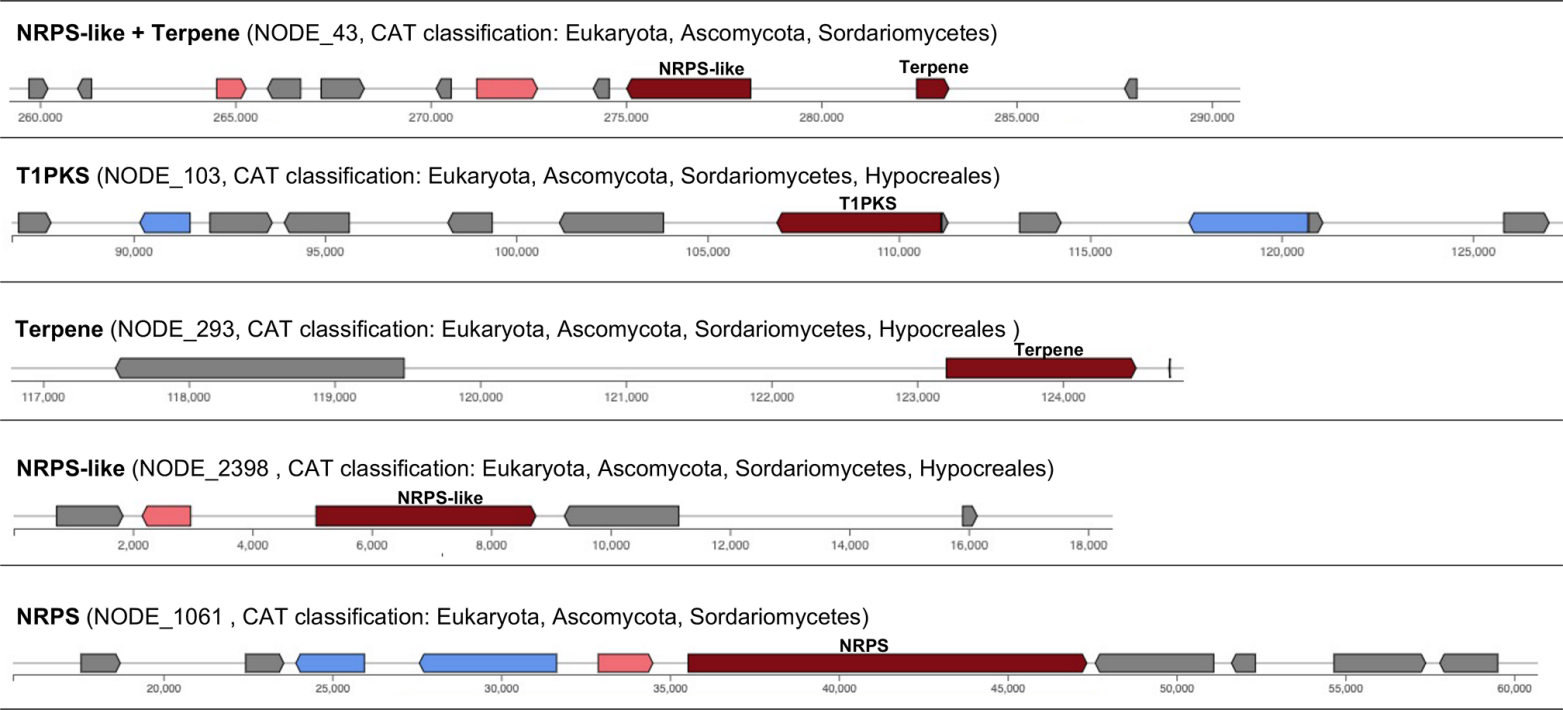
Four BGCs on beet endophyte metagenome contigs classified as eukaryotic by Whokaryote. These five BGCs were predicted by fungiSMASH, which uses GlimmerHMM as a gene predictor, but were not found with antiSMASH, which uses Prodigal-M as a gene predictor.

We also ran antiSMASH and fungiSMASH (version 5.1.2) on the sugar beet endophyte metagenome (Supplementary Material S5). AntiSMASH found 28 BGCs on eukaryotic contigs, while fungiSMASH found 24 BGCs on eukaryotic contigs. FungiSMASH reported five BGCs on eukaryotic contigs that were not found by antiSMASH, namely a type one polyketide synthase (T1PKS) gene cluster, a terpene BGC, a non-ribosomal peptide synthetase (NRPS) gene cluster, an NRPS-like gene cluster, and a hybrid-type cluster classified as both 'NRPS-like' and 'terpene' (Fig. 3). Additionally, fungiSMASH found 12 BGCs on prokaryotic contigs that were not found by antiSMASH. At the same time, there were also eight BGCs on eukaryotic contigs that were only predicted by antiSMASH and not by fungiSMASH. This shows that simply using a general eukaryote-specific gene predictor (GlimmerHMM) that was trained on a single species (as implemented in antiSMASH) on eukaryotic contigs does not necessarily lead to better gene predictions for BGC detection. BGC core genes, which are scanned for signature domains, are usually very large and may contain multiple domains. Possibly these genes are more difficult to predict correctly for eukaryotic gene finders. Nevertheless, some new BGCs were found that would not have been found with bacterial antiSMASH. Therefore, we suggest using both Prodigal and eukaryotic gene predictors (e.g. GlimmerHMM [23], Augustus [13], and/or MetaEuk [15]) to predict genes on standalone eukaryotic contigs and using both annotations for further functional analyses. Previous findings showed that using a eukaryotic gene predictor on a metagenome-recovered eukaryotic bin resulted in a more complete predicted gene set when compared to MetaProdigal predictions [14]. Our results show that repredicting genes on unbinned contigs is also worthwhile and can lead to the discovery of genes and functions that would not have been found otherwise. Future research should focus on more accurate *ab initio* eukaryotic gene prediction on unbinned contigs to prevent these extra steps.

## Conclusions

Studying eukaryotes in metagenomes is still challenging, and tools that aid with this process are scarce and not widely used yet. We show that manually selected features based on fundamental differences in gene structure between eukaryotes and prokaryotes can be used to reliably classify metagenomic contigs as eukaryotic or prokaryotic. Using only these features leads to slightly worse performance compared to k-mer frequency-based approaches. With Whokaryote+Tiara, we combined our selected features with the output from the k-mer based deep learning classifier Tiara. The resulting classifier achieves nearly flawless classification of contigs from a wide range of organisms and compensates biases present in the individual classifiers. Contigs, including unbinned ones, predicted as eukaryotic can be included in metagenomic pipelines using eukaryote-specific tools, allowing new discoveries about their roles in microbiomes.

## Supplementary Data

Supplementary material 1

Supplementary material 2
