# Supplementary material for: Whokaryote: distinguishing eukaryotic and prokaryotic contigs in metagenomes based on gene structure
Source: Microb Genom. 2022 May 3;8(5):mgen000823. doi: 10.1099/mgen.0.000823 (PMC9465069; doi:10.1099/mgen.0.000823)
Supplement: Supplementary material 1 [file mgen-8-823-s001.pdf]

Supplementary Figures

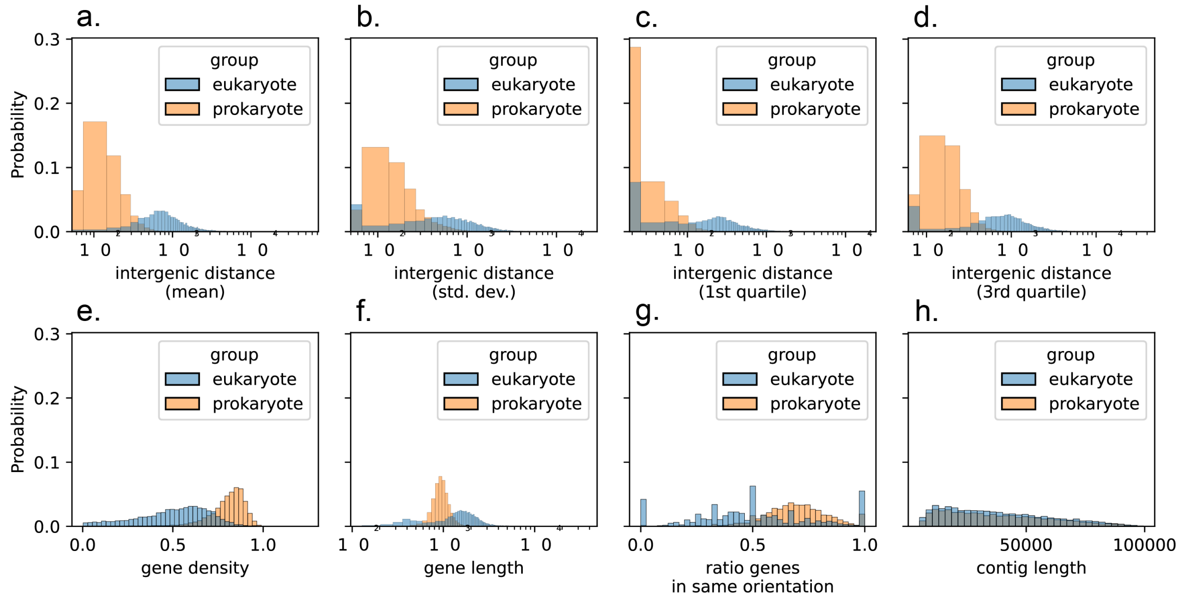

Supplementary Figure 1. Overview statistics of the features calculated for reference gene annotations downloaded from NCBI corresponding to the artificial contigs from 87 out of 99 genomes in the training dataset. The probability distribution of the feature values of all contigs are shown, grouped by the taxonomic group (eukaryote or prokaryote) the contig belongs to. For every contig, the mean, standard deviation, first quartile and third quartile of the intergenic distances between the genes was calculated (a-d, respectively). e: The mean gene density of every contig, calculated by dividing the sum of the length (in base pairs) of all genes by the total length of the contig. f: The mean gene length of every contig, calculated as the end position of the gene minus the start position. g: The ratio of genes on a contig that are on the same strand (e.g., they have the same 'orientation'). h. The distribution of contig lengths.

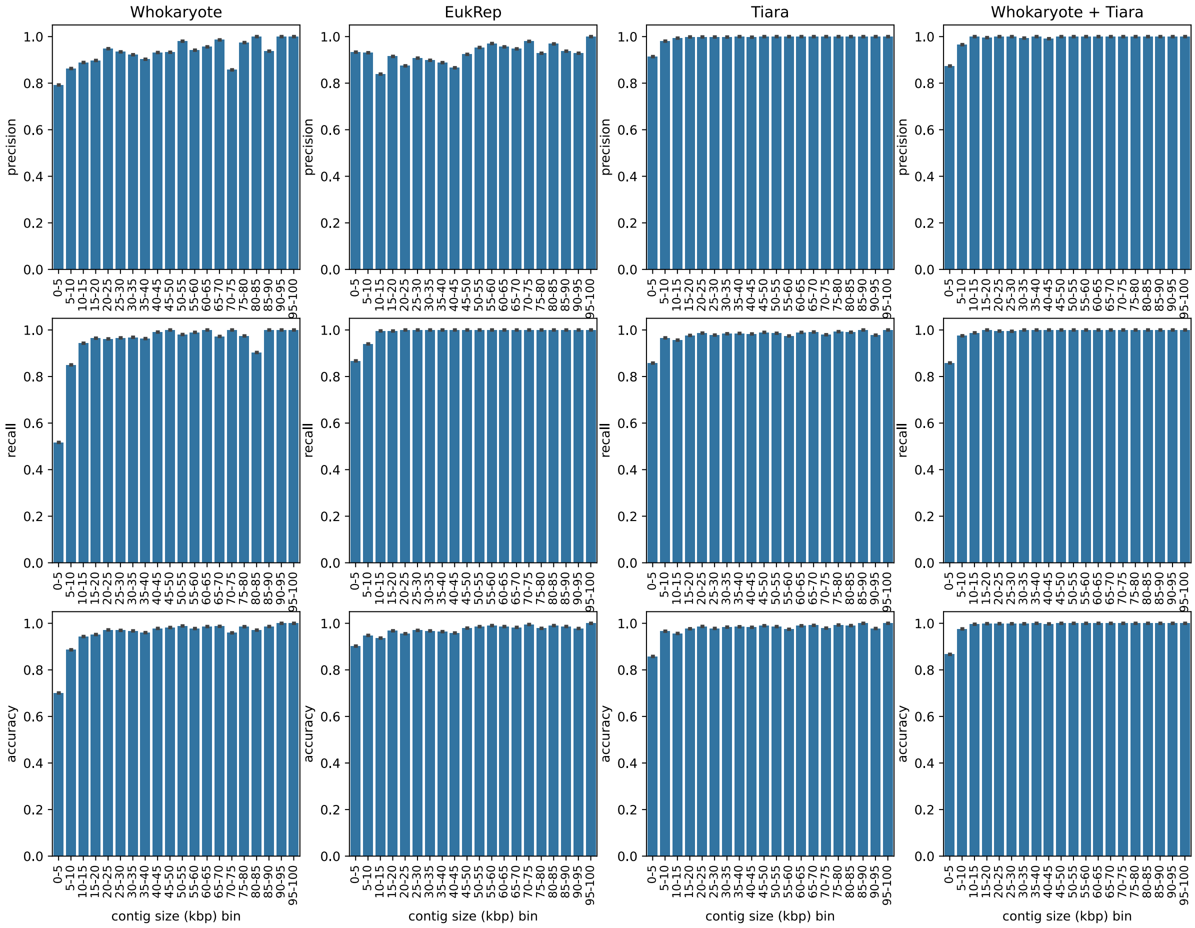

Supplementary Figure 2. The classification accuracy on the test dataset (14 eukaryotes and 25 prokaryotes) in different contig length categories of EukRep, Tiara, Whokaryote, and Whokaryote + Tiara, shown as relative count of incorrect and correct predictions.

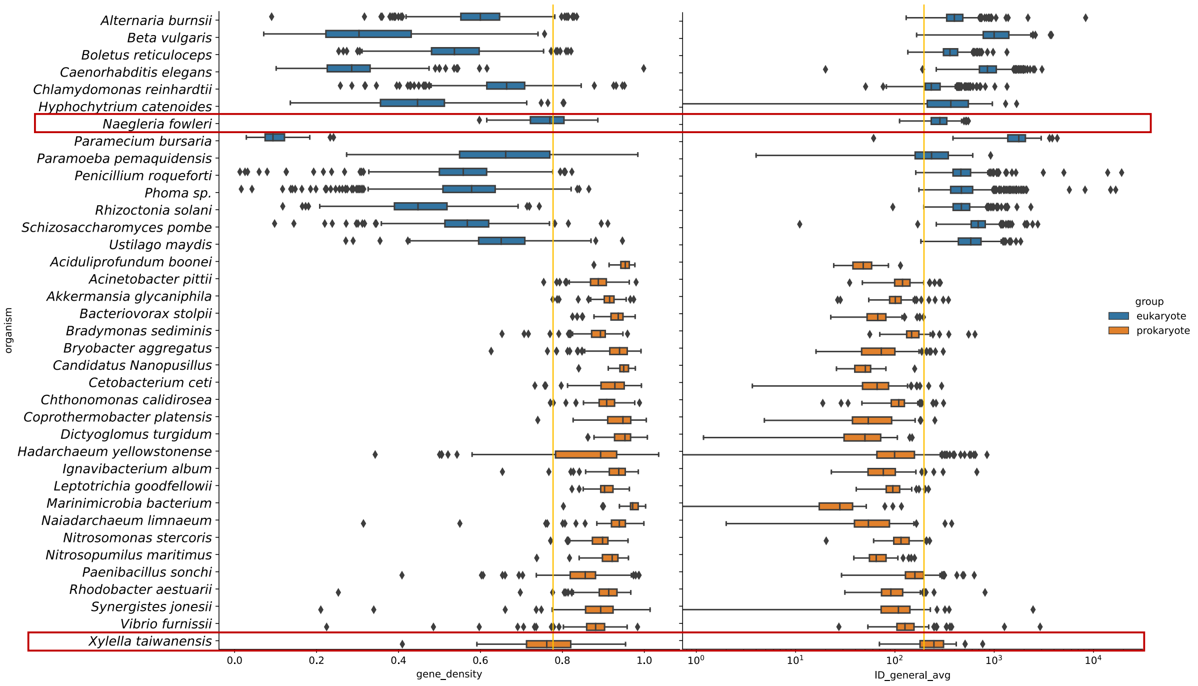

Supplementary Figure 3. On the left, the distribution of gene density and average intergenic distances per contig is shown per organism. A vertical yellow line was drawn roughly between the eukaryotic and prokaryotic boxes. A red box is drawn around the results for Naegleria fowleri, a eukaryote with a gene density and intergenic more typical for prokaryotes, and Xylella taiwanensis, a prokaryote with a gene density and average intergenic distance of its contigs very close to the range of most eukaryotes. The yellow line is drawn to roughly indicate the value that separates the majority of eukaryotes and prokaryotes.

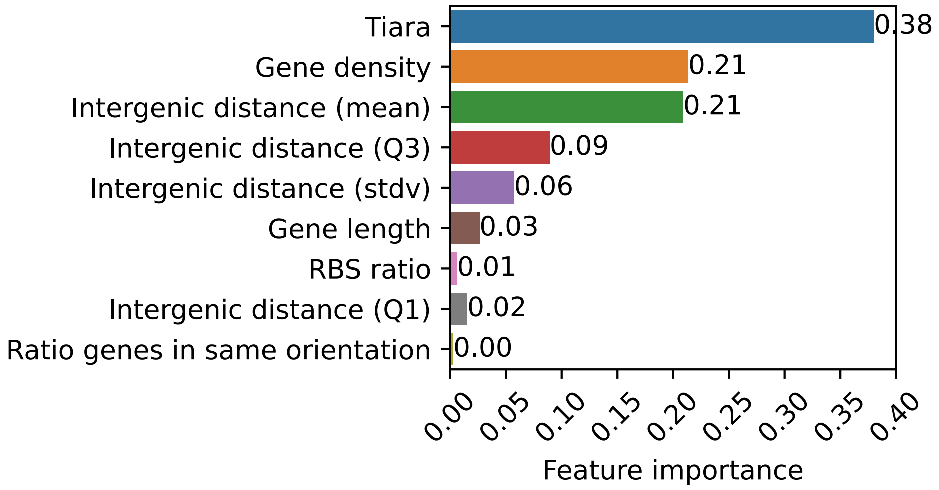

Supplementary Figure 4. Feature importance of the enhanced random forest classifier 'Whokaryote', which uses Tiara predictions (eukaryote/prokaryote) as an extra feature. The classifier was trained on a dataset of 99 eukaryotic and prokaryotic reference genomes that were split into artificial contigs of a length between 5kbp and 100kbp.

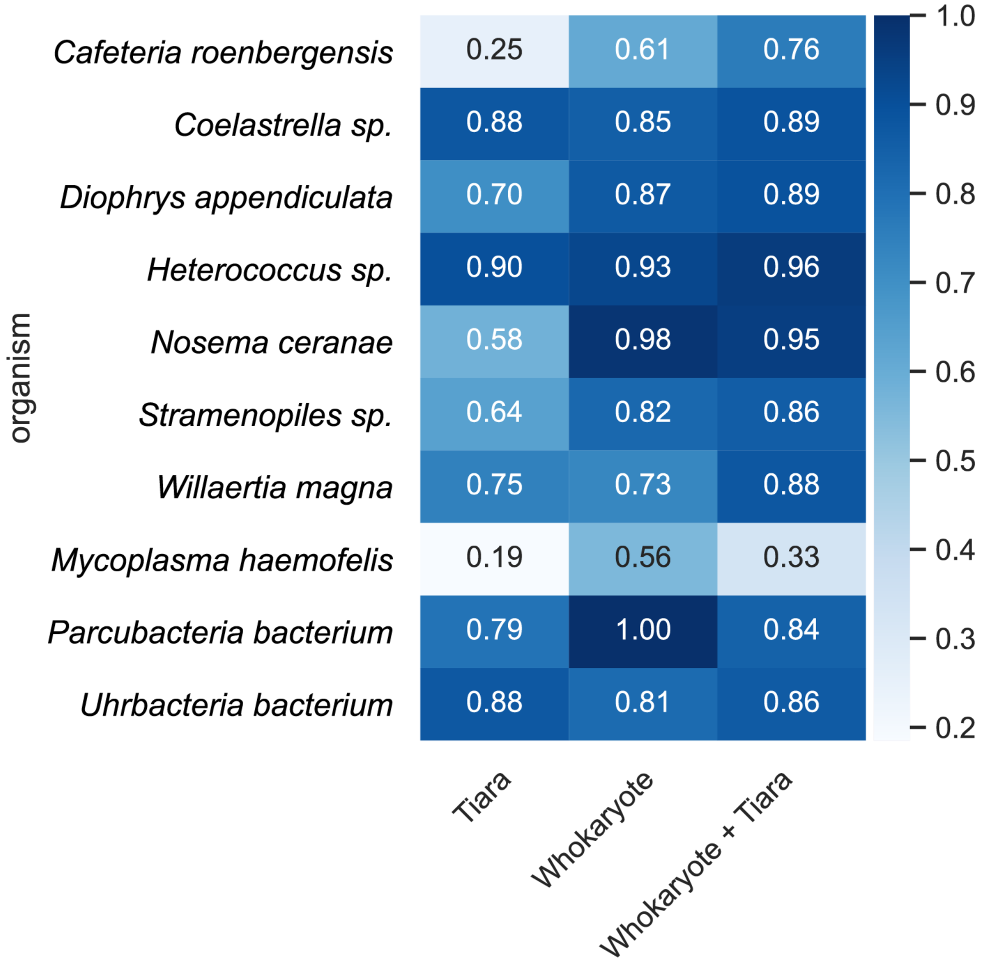

Supplementary Figure 5. Tiara, Whokaryote, and Whokaryote + Tiara were tested on a set of genomes on which Tiara performed relatively badly. The genomes of these organisms were split into random contigs between 5kbp and 100kbp with a total of around 1600 contigs.
